# Mosquito-Borne Viruses and Non-Human Vertebrates in Australia: A Review

**DOI:** 10.3390/v13020265

**Published:** 2021-02-09

**Authors:** Oselyne T. W. Ong, Eloise B. Skinner, Brian J. Johnson, Julie M. Old

**Affiliations:** 1 Children’s Medical Research Institute, Westmead, NSW 2145, Australia; oong@cmri.org.au; 2 Mosquito Control Laboratory, QIMR Berghofer Medical Research Institute, Herston, QLD 4006, Australia; Brian.Johnson@qimrberghofer.edu.au; 3 Environmental Futures Research Institute, Griffith University, Gold Coast, QLD 4222, Australia; ebskinn@stanford.edu; 4 Biology Department, Stanford University, Stanford, CA 94305, USA; 5 School of Science, Western Sydney University, Hawkesbury, Locked bag 1797, Penrith, NSW 2751, Australia

**Keywords:** mosquito vectors, arboviruses, disease reservoirs, animal diseases, animal distribution

## Abstract

Mosquito-borne viruses are well recognized as a global public health burden amongst humans, but the effects on non-human vertebrates is rarely reported. Australia, houses a number of endemic mosquito-borne viruses, such as Ross River virus, Barmah Forest virus, and Murray Valley encephalitis virus. In this review, we synthesize the current state of mosquito-borne viruses impacting non-human vertebrates in Australia, including diseases that could be introduced due to local mosquito distribution. Given the unique island biogeography of Australia and the endemism of vertebrate species (including macropods and monotremes), Australia is highly susceptible to foreign mosquito species becoming established, and mosquito-borne viruses becoming endemic alongside novel reservoirs. For each virus, we summarize the known geographic distribution, mosquito vectors, vertebrate hosts, clinical signs and treatments, and highlight the importance of including non-human vertebrates in the assessment of future disease outbreaks. The mosquito-borne viruses discussed can impact wildlife, livestock, and companion animals, causing significant changes to Australian ecology and economy. The complex nature of mosquito-borne disease, and challenges in assessing the impacts to non-human vertebrate species, makes this an important topic to periodically review.

## 1. Introduction

Mosquito-borne diseases pose a great risk to public health threatening more than half the human population, and many non-human vertebrates [1]. In Australia, mosquito-borne diseases are currently a major focus due to public health concerns, which have been amplified by the potential effects of climate change and urbanization (see [2] for a review). Exacerbating the effects of climate change is the rapid and sustained increase in global trade and the alteration of the physical environment as a result of urbanization, deforestation, and agricultural expansion. Such changes have been highly favorable to urban vectors of foreign viruses including dengue, chikungunya, and Zika virus [3], whereas increased air travel and trade has led to a proliferation of viruses and their vectors globally [4,5]. However, the impacts of these global phenomena on non-human vertebrate hosts (species which may be susceptible to infection) and the consequences to the maintenance and transmission of mosquito-borne viruses remains poorly understood.

Australia has a diverse climatic range and environmental bioregions, which promote unique and endemic faunal diversity. This, in combination with a number of introduced vectors and pathogens, has resulted in the emergence of novel disease transmission pathways, including those of regional and global importance. Additionally, due to the dispersal of Australian animals, distinct patterns in evolution of mosquito-borne viruses have been found. The viruses across Australia either (i) evolve slowly and uniformly, (ii) have significant divergence due to single nucleotide changes, or (iii) have multiple lineages that are periodically redistributed over the Australian continent [6]. While studies on the distribution of mosquitoes and human infection rates are important, it is essential to consider the complex relationship between vectors and non-human vertebrates in various regions throughout Australia. Here, we review evidence for the impacts of Australian mosquito-borne viruses (introduced and endemic) on non-human vertebrates (including native and domestic species), and the role that these vertebrates may play in disease and transmission cycles. The aim of this review is to assess the impact of mosquito-borne diseases to Australian animals and the implications of the rapid change in Australia due to urbanization and climate change. We conclude with a discussion of exotic mosquito-borne diseases that threaten the unique fauna of Australia.

## 2. Mosquitoes: The Link between Vertebrate Host and Disease

There are more than 300 species of mosquitoes identified in Australia and almost 100 of these are capable of transmitting pathogens to wildlife and domestic animals (see Table 1), of which *Aedes*, *Culex*, and *Anopheles* species are the most common genera of vectors [7]. Feeding preferences by these species are highly variable with some species (such as *Aedes aegypti*) reported to have host specific feeding patterns, while other species (such as *Aedes vigilax*) exhibit more generalist feeding behaviors [8], both of which can play an important role as bridging vectors [9,10]. Individual feeding patterns are often dependent on host abundance and availability, both of which are strongly linked to habitat identity, and which can change annually and seasonally depending on the biology and ecology of individual host species (see [11] for a review). To date, vertebrate blood-meal hosts have been identified for a variety of taxonomic groups, including Carnivora (e.g., cats, dogs, and foxes), Aves (birds), Diprotodontia (e.g., possums and macropods), Artiodactyla (e.g., cattle, sheep, pigs, and goats), and Equidae (horses), with individual vector species displaying a trade-off between host preference and host availability [11]. For example, in rural Queensland, Australia, bloodmeal origins for *Culex annulirostris*, were dominated by cattle [12], but in Sydney, a highly urbanized city, *Cx. annulirostris* bloodmeal origins were mostly from birds, rodents and rabbits [8]. Thus, the ecologies of Australian mosquito-borne viruses can be driven by complex interactions between vector species, host availability, and host preference, which may be a derivative of habitat or climate. This will ultimately drive the spread of pathogens.

## 3. Uniquely Australian Vertebrate Hosts

**Figure 1:**
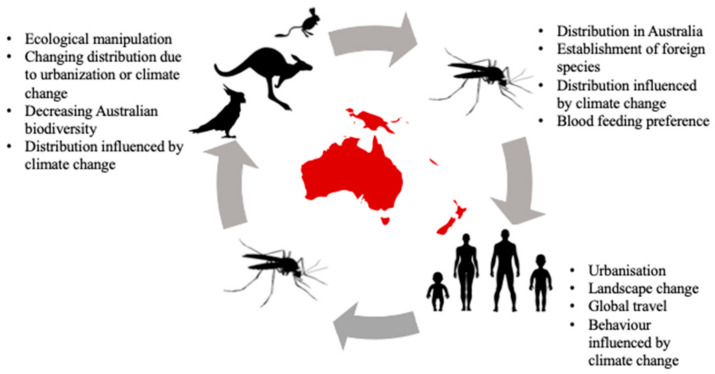
Relationship between Australian wildlife, mosquitoes, and humans. Often mosquito-borne diseases are often spread from human to human via the bite of a mosquito; however, consideration of Australian wildlife is important as they are often hosts for these pathogens. There are also many factors that can determine the spread of mosquito-borne disease, including the change in wildlife distribution, which should be considered when developing strategies to minimize the spread of disease.

Australia provides a unique opportunity to investigate the transmission of viruses between vertebrates and mosquitoes. The long geographic isolation of Australia has led to the co-evolution of viruses, mosquitoes, and endemic vertebrate hosts, which offer unique insights into immunology and physiology. Over the last 200 years, Australia has also experienced the introduction of viruses and domestic species, and an expansion of urbanization, all of which have shifted the dynamics of disease and ecology among native species. This crockpot of co-evolution and introductions mean that Australian fauna have highly heterogenic roles for transmitting different viruses within the community (Figure 1), or are affected by viruses in different ways (i.e., asymptomatically vs. symptomatically; Table 1).

### Marsupials

There are more than 300 extant marsupial species globally, of which close to 70% occur on the Australian continent (the mainland, Tasmania, New Guinea, and nearby islands), representing the most diverse extant marsupial radiation [71]. Many species exhibit unique physiological characteristics, such as adaptations to specific climatic envelopes, which have allowed them to succeed in even the harshest of Australian environments. However, immunological characteristics of marsupials may increase their susceptibility to infection for mosquito-borne viruses. When compared to eutherian mammals, neonatal marsupials are born without histological mature immune tissues [72,73] and are therefore unable to mount specific immune responses and are presumably highly reliant on maternal and innate immune strategies. Furthermore, some studies have reported marsupial immune systems are slower to mount some specific immune responses and occur at lower levels than those mounted by eutherian species [74,75]. However, the impact of such differences on disease susceptibility are poorly understood and the many similarities between eutherian and marsupial immune systems [76] cannot be overlooked (Figure 1).

The immediate threat of land use and climate change on the survival of many Australian marsupial species highlights a need to better understand the impacts disease on marsupial health and reproductive fitness. Additionally, we need to consider the ecological impacts of exotic mosquito introductions and range expansions on native species. The proliferation of global trade and travel makes the introduction exotic mosquito species highly likely (see [77,78] for reviews), whereas climate change is already changing native species distributions [3].

## 4. Ross River Virus

Ross River virus (RRV; belonging to the family Togaviridae and genus *Alphavirus*) is the most common arboviral disease in Australia [79,80,81]. Human infection can lead to chronic polyarthritis, with some symptoms lasting more than a year [82]. Although predominantly endemic to Australia, emerging evidence suggests RRV circulation is occurring in Pacific Island countries, including Fiji [83], American Samoa [84], Cook Islands [85], and French Polynesia [86]. Identifying the cause of RRV outbreaks is critical in Australia, as human infections are reported nationwide. Australia has approximately 5000 RRV human infection cases reported annually [23]. RRV is maintained in the environment through complicated transmission dynamics with multiple vectors and hosts. Since it was first identified in 1948, RRV has been isolated from more than 40 different mosquito species [87]. Under laboratory investigations the most prominent vectors are thought to be *Aedes camptorhynchus*, *Aedes notoscriptus*, *Ae. Vigilax*, and *Cx. annulirostris* based on their ability to amplify RRV in their saliva and transmit RRV in mouse-models [19,88].

Of the 81 non-human vertebrate species that have been serologically tested in Australia, 60 have had positive antibodies to RRV including domestic and livestock species including dogs (*Canis lupus familiaris*), cattle (*Bos taurus*), and horses (*Equus caballus*), and native birds, marsupials (possums and macropods), and flying foxes (*Pteropus* spp.) [20]. Recently, a sero-survey of koalas in Queensland, Australia found more than 80% have been exposed to RRV [89]. Not all of these species are thought to be important as reservoirs of RRV. Under experimental infection conditions, marsupials develop one of the highest-longest lasting viraemias [90]. Given the extensive length of marsupial viremias, it formed the basis of a long-held dogma that marsupials are better reservoirs than eutherian mammals and birds. The absence of marsupials in some Pacific Islands where local transmission of RRV is reported suggests that other species likely act as reservoirs. There is evidence that suggests birds may be important. Firstly, experimentally infected little corellas (*Cacatua sanguinea*) infected 14% of susceptible mosquito vectors, despite developing a relatively low-short lived viraemia [90]. Secondly, the first isolates of RRV from three bird species, magpie larks (*Grallina cyanoleuca*), Australian brown flycatcher (*Microeca leucophaea*), and masked finch (*Poephila personata*) [19], demonstrating that the virus circulates among bird populations. Overall, RRV in birds has been largely understudied and requires further investigation. Another understudied, but potential reservoirs of RRV are murids. Murids demonstrate moderate viraemia under experimental infection conditions [90] and one modelling study found house mouse (*Mus musculus*) abundance closely correlated with human notifications of RRV in Victoria [91].

Horses are the only species other than humans that have been reported to have clinical symptoms associated with RRV, including joint swelling and muscle stiffness [92]. Serological surveys of horses in Australia have varied between 26% [21] and 91% [93] being seropositive for RRV. Horse populations in Australia are estimated to exceed 1.2 million individuals and the thoroughbred industry was estimated to be $6.3 billion alone in 2001 [94]. In 2011, a national outbreak of equine diseases show high RRV infections in horses showing neurological or muscular symptoms [22]. As such, RRV can have large economic and ethical implications for horses in Australia and potentially internationally. Further studies are needed to determine the true burden of RRV in horse populations, and to develop appropriate treatments and preventative measures.

Future investigations of RRV in non-human vertebrates would benefit from additional field surveillance to identify ecological traits (such as habitat, seasonality, feeding behavior, and reproduction) that may be important for ongoing transmission [95,96]. There is some evidence to suggest that landscapes with proximity close to water reservoirs and the presence of some marsupial reservoirs is considered high risk for RRV disease transmission [97]. Further, animals with longer gestation periods, have dietary specialization and small population density are also more likely to have a history of RRV infection, suggesting that these animals should be monitored prior to an outbreak [98]. More localised research is needed to determine the significance of these trends in a given area.

## 5. Barmah Forest Virus

Barmah Forest virus (BFV) is a zoonotic alphavirus with humans infections reported nationally. In humans, BFV presents with similar clinical signs as RRV including polyarthritis, rash, fever, and myalgia [99]. BFV was first isolated from *Cx. annulirostris* mosquitoes in northern Victoria in 1974 [100]. However, given the similarity in clinical symptoms to RRV [80], it is likely that human cases of BFV may have been previously misdiagnosed and believed to be RRV. On average, there are 2400 notifications of BFV in Australia annually, with the majority of cases reported in Queensland [23]. The 2012–2013 period marked the largest BFV epidemic on record in Australia, with more than 2223 notifications in Queensland and 1024 notifications in Western Australia [23].

BFV has been isolated from a number of wild-caught mosquito species, including *Cx. annulirostris* [101] and *Ae. vigilax* [101,102,103,104]. Other insect vectors include the biting midge (*Culicoides marksi*) [105,106,107]. Vector competence studies found that an urban freshwater species, *Ae. notoscriptus*, was moderately susceptible to infection with transmission occurring between days 5 and 12, and an average transmission rate of 45% [108]. *Ae. vigilax* and *Aedes procax* have also demonstrated a high susceptibility to infection under vector competence studies [24], but *Cx. annulirostris* is a relatively ineffective vector of BFV with infection not exceeding 8% [109].

Evidence for BFV transmission in non-human vertebrates is limited. Serological investigations have found exposure of BFV in a diversity of non-human vertebrates. Moderate exposure has been reported in eastern grey kangaroos (*Macropus giganteus*) (44%) [25] and cattle (29%) [110], and low seropositivity in common brushtail possums (*Trichosurus vulpecula*) (10.7%) [21], koalas (*Phascolarctos cinereus*) (9%) [25], quokkas (*Setonix brachyurus*) (3.2%), domestic cats (*Felis catus*) (2%) [27], domestic dogs (*Canis lupus familiaris*) (1.3%) [27], and horses (1.2%) [70]. Despite a relatively large number of bush rats (*Rattus fuscipes*) and swamp rats (*Rattus lutreolus*) being tested, no exposure to BFV was found in these species [26]. Only common brushtail possums, dogs and cats have been experimentally infected with BFV, all of which demonstrated poor capability as amplifiers. Two of the 10 possums developed an immune response to the infection and had detectable antibodies for at least 45 days following the infection, however the species did not develop sufficient viraemia to infect susceptible mosquitoes [42]. Similarly, none of the 10 dogs or cats developed a detectable viraemia for BFV, and just one dog and three cats developed antibodies post infection [111]. Future serological, experimental and modelling studies on the non-human vertebrates of BFV would greatly improve current understandings for this medically important arbovirus.

## 6. Sindbis Virus

Sindbis virus (SINV) is one of the most commonly isolated arboviruses in Australian mosquitoes, despite rare instances of human infection. There are two different genotypes of the Sindbis virus: (i) the Oriental/Australian strain circulating throughout most of Australia (excluding Tasmania) and other surrounding countries including Malaysia and Papua New Guinea; and (ii) a strain endemic to southwestern Australia, with the Oriental/Australian SINV strain first isolated in 1960 from *Cx. annulirostris* in far north Queensland [28]. More recently, a new strain of SINV endemic to the south-west region of Western Australia, which differs in nucleotide sequences from the Oriental/Australian strain and Paleoarctic/Ethiopian strain by 25.4–28.9% and 16.8–19.4%, respectively [30]. The higher similarity between the endemic south-west Western Australia isolates and Paleoartic/Ethiopian strain suggested that this particular strain was imported by a traveler or migratory bird, and selective pressures in that region resulted in a new SINV strain.

Isolations of SINV have been found *in Cx. annulirostris*, *Aedes normanensis*, *Ae. camptorhynchus* [29], and *Aedes pseudonormanensis* [30]. There is evidence for the vertical transmission of SINV, particularly in *Ae. camptorhynchus* [29]. It has been reported that SINV infection is higher in *Ae. aegypti* mosquitoes that were reared at 30 °C compared to 20 °C [112], suggesting that warmer temperatures as a result of climate change could potentially increase SINV transmission.

Migratory birds are considered the main amplifying host for SINV, particularly those that have migration patterns connecting Australia [30], United Kingdom [113], northern Europe [114], South Africa [115], and China [116]. Birds that are vectors for SINV have persistent infections without any clinical symptoms, and are therefore healthy and able to travel between countries [113]. Although birds are considered one of the primary vectors for SINV, they do not necessarily contribute to outbreaks [116]. Additionally, many endemic birds found in south-west Western Australia are sedentary, suggesting that the new south-west Western Australia isolate is maintained through birds that do not travel long distances or vertebrate hosts available in its surrounding areas which includes marsupials [30]. Antibodies to SINV have been found present in one chuditch (*Dasyurus geoffroi*), emus (*Dromaius novaehollandiae*), European rabbits (*Oryctolagus cuniculus*), and horses in Australia [31].

## 7. Murray Valley Encephalitis Virus

Murray Valley encephalitis virus (MVEV) human infections are often asymptomatic, however approximately 1:150 to 1:1000 MVEV infections result in symptomatic encephalitic disease, which may include neurological features [117,118]. Although cases of MVEV in humans are not common, its high mortality/morbidity rate relative to other circulating flaviviruses is cause for concern when outbreaks occur. Several large outbreaks and epidemics have been reported since the early 20th century following its isolation from a human in 1951 [118]. To date, cases of MVEV have been reported in most Australian states, with four major outbreaks on the east coast, including two human MVEV isolations in Papua New Guinea in 1956 [119] and 1960 [120]. Despite this large historical distribution, MVEV is only considered endemic across northern Australia and Papua New Guinea [118].

MVEV has been isolated from *Culex sitiens* and other *Culicine* mosquitoes [36] but the major vector implicated in MVEV transmission is *Cx. annulirostris* [35]. Vector competence studies found that *Cx. annulirostris* from two different colonies (Queensland and Victoria) have been shown to transmit MVEV at a 75–100% success rate, even at temperatures as low as 20 °C [121]. *Culex pipiens quinquefasciatus* has also been assessed as a potential vector for MVEV, but had a poor average infection rate of 12.9% [122].

Mammalian species likely play an important role in the secondary transmission MVEV. MVEV was implicated in the 2011 national equine outbreak, though the number of infections is less compared to RRV and West Nile virus (WNV) [22]. That same year, seroconversion in sentinel chickens along Murray River was detected after high rainfall and flooding, indicating an increased risk of MVEV infections [117]. Of the currently investigated marsupial species, western grey kangaroos (*Macropus fuliginosus*) may play a role as important reservoirs as they develop sufficient viraemia to infect *Cx. annulirostris* mosquitoes, whereas agile wallabies (*Macropus agilis*) do not as they do not develop high enough or long lasting viraemias [37]. MVEV transmission without detectable viremia in several other marsupial species may occur although it is unknown whether this contributes significantly to the maintenance of MVEV in the wild [37].

Following a major outbreak of MVEV in the Murray Valley in 1951, serological investigations were undertaken for a number of other wild and domestic vertebrate species [123,124]. These early investigations found that waterbirds are commonly infected with MVEV. Further serological investigations in waterbirds following a 1974 outbreak of MVEV in south-western New South Wales and northern Victoria found that *Ciconiiformes*, particularly rufous night herons (*Nycticorax caledonicus*), had the highest seropositivity rate (55%) [34]. Australian avian species are also implicated in the transmission of MVEV, including galahs (*Eolophus roseicapilla*), sulphur-crested cockatoos (*Cacatua galerita*), and Pacific black ducks (*Anas superciliosis*). These bird species can develop a moderate viraemia lasting 1–9 days and infect up to 50% of susceptible *Cx. annulirostris* following a blood meal [38]. Thus, MVEV is speculated to be maintained in an enzootic cycle largely involving waterfowl and ornithophilic mosquitoes in the north of Western Australia and the Top End of the Northern Territory [118,125]. However, the importance of marsupials and other non-avian vertebrates in the transmission of MVEV warrants further investigation.

## 8. West Nile Virus

West Nile virus (WNV) was first isolated in 1937 in Uganda, Africa, and currently circulates throughout the Americas, Europe, and Asia [126]. The virus is thought to have been introduced into Australia, possibly through travellers from Europe and the transportation of convicts [127]. WNV Kunjin strain (WNV_KUN_) is endemic to tropical northern Australia and was first isolated from *Cx. annulirostris* in 1960 in northern Queensland [128]. Overall, WNV_KUN_ has been isolated from all Australian states [42,43] and is especially prevalent around tropical areas in northern Queensland and the Northern Territory [62], though incidents of the disease are rarely reported in humans. WNV_KUN_ has consistently been isolated from *Cx. annulirostris* since 1984 and is found in all states in Australia [44], but the virus has also been isolated from *Cx. australicus*, *Cx squamosus*, *Cx. quinquefasciatus*, *Ae. tremulus*, *Ae. alternans*, *Ae. nomenensis*, *Ae. Vigilax*, and *Anopheles amictus* [45,46].

In 2011, a new strain of WNV_KUN_, WNV_KUN2011_, was characterized due to an unprecedented outbreak in horses [42]. Clinical signs were recorded in more than 1000 horses with a fatality rate of 10–15% [42]. Previous WNV_KUN_ infections usually occurred when flooding occurs due to high rainfall that supported mosquito and waterbird populations; however, even though high rainfall did occur during the outbreak, mosquito populations remained small in many of the affected areas suggesting that WNV_KUN2011_ is more virulent compared to WNV_KUN_ [22,42]. Additionally, it was found that *Cx. annulirostris* transmitted WNV_KUN2011_ more efficiently compared to other WNV_KUN_ strains [48].

Historically, mammals have been thought to be dead-end hosts because of their short-term, low viremia [129]. However, more recent studies have shown that this may not be the case with some wild mammals having high seroprevalence. In the United States, urban mosquitoes such as *Aedes albopictus* and wild mammals are increasingly implicated in maintenance of WNV and may be establishing a unique transmission cycle that does not involve birds [130,131,132]. Wild mammals implicated in WNV transmission include the Virginian opossum (*Didelphis virginiana*), certain species of tree squirrel (*Sciurus* spp.), eastern chipmunks (*Tamias striatus*), and eastern cottontail rabbits (*Sylvilagus floridanus*) [133,134,135,136]. Although these overseas studies have not investigated the Australian strain, WNV_KUN_, it is important to be aware of these transmission dynamics, as Australian mammals could be used as indicators for future transmission studies should overseas strains enter Australia.

It is well-known that WNV is spread by migratory birds (see [43] for a review), with the rufous night heron considered one of the main reservoirs [137], however a study found one Australian white ibis (*Threskiornis moluccus*) had antibodies to the virus [47]. Australian white ibis are common in urban areas around Australia, which suggests that they could be effective reservoirs for zoonotic pathogens. WNV_KUN_ have been exposed to house sparrows (*Passer domesticus*), however, was shown to be non-virulent as opposed to the West Nile virus Afro-European and North American strain [49]. With no other comparative viremia profiles among urban Australian birds, the ibis should be considered a potential host in future studies. The role of other Australian fauna in the transmission WNV_KUN_ is poorly understood and further investigations are necessary.

## 9. Japanese Encephalitis Virus

Japanese Encephalitis virus (JEV) is an acute arbovirus disease associated with encephalitis. The first outbreak was detected in Torres Strait in 1995 after the viral isolation of two Badu Island residents. *Cx. annulirostris* was implicated as the major vector for the JEV outbreak as mosquito surveys revealed that their populations bred near the Badu community in pigpens and JEV isolations were only found in *Cx. annulirostris* at the time [53]. Enzootic transmission is suspected to occur between migratory birds, frugivorous bats, or mosquitoes [53,138]. Most humans exhibit little to no symptoms, but some can develop encephalitis and 25% of these cases can be fatal [139], making JEV a significant public health threat where endemic.

In northern Australia and Papua New Guinea, JEV occurs sporadically due to migratory birds, travelling mosquitoes, and the close proximity of domestic pigs to humans [53,54]. Domestic pigs are considered one of the main reservoirs for JEV, alongside frugivorous bats that are suspected to be involved in the introduction of JEV into Australia [138], particularly because they have high titres of JEV antibodies in other countries [140]. Black flying foxes (*Pteropus alecto*) are common throughout north and eastern Australia [141]. Experimental JEV inoculations in black flying fox resulted in no JEV symptoms and only one (out of five) inoculated individuals showed low-level viremia, despite detection of anti-JEV antibodies in all individuals. While little to no viraemia were detected in all individuals infected, some were still capable of infecting *Cx. annulirostris*, suggesting the potential of black flying fox populations to cause JEV outbreaks [60]. As these bats show no detectable viremia but still infect vectors it suggests that their potential role in the transmission of JEV should be more thoroughly investigated. Moreover as bats are known hosts of many other viruses that are pathogenic to a wide range of mammals including humans, and exhibit no clinical symptoms, future studies should further investigate bats as potential reservoirs for JEV and other arboviruses (see [142,143] for reviews). Furthermore, additional studies including the other three species of Australian flying fox, the little red (*Pteropus scapulatus*), spectacled (*P. conspicillatus*), and grey-headed (*P. poliocephalus*) may provide insights into the potential of future viral outbreaks given the wide distribution of flying foxes in northern and eastern Australia, as observed in studies on Hendra virus [144]. This includes areas where the distribution of competent vector species and flying foxes overlap, particularly given bats can transmit JEV with no detectable viremia [138]. Studies should also investigate flying foxes, and other potential reservoir species, in terms of physiological stress. For example, McMichael, et al. [145] indicated an indirect association between lower temperatures and physiological stress in black flying foxes and increased Hendra virus infection and excretion. These factors may also have implications for arbovirus outbreaks due to reservoir species biology.

## 10. Kokobera and Related Viruses

There are currently five known members of the Kokobera virus (KOKV) group which are native to Australia [128]. These include the KOKV (isolated in 1960, Queensland), Stratford (STRV) (isolated in 1961, Cairns), New Mapoon (NMV) (isolated in 1998, Cape York Peninsula), Bainyik (BAIV) (previously strain MK7979; isolated in 1966, Papua New Guinea), and Torres (previously strain TS5273; isolated in 2000, Torres Strait) viruses [146]. Compared to the prototype strain (KOKV), other members in the Kokobera virus group have different antigenic profiles based on their monoclonal antibody binding patterns [146,147].

Altogether, the KOKV group has many different mosquito vectors. STRV alone has been isolated in six mosquito vectors, which include five *Aedes* spp. (*Aedes aculeatus*, *Ae. alternans*, *Ae. notoscriptus*, *Aedes procax*, and *Ae. vigilax*) and *Anopheles annulipes* [63]. STRV was first isolated in *Ae. vigilax* from Cairns [90]. Similar to KOKV, the NMV was also first isolated from *Cx. annulirostris* [146]. BAIV and Torres virus were isolated from a pool of mosquitoes [148,149].

An experimental study found that only BAIV produced signs of encephalitis in mice, and while virus were detected up to day 3 post-infection, hardly any were detected in the organs of the experimental mice [65]. When inoculated directly into mouse brain, all viruses in the Kokobera virus group (except NMV) caused mortality in mice suggesting the possibility of replication in the brain and high neurovirulence [65]. Antibodies to KOKV have been found in kangaroos, horses, cattle, and wallabies in Australia [17,64]. While considered endemic to certain regions of Australia, KOKV antibodies have previously been found in Indonesian cattle from Java and Bali [150]. Marsupials (specifically macropods) are suspected to be an important reservoir for KOKV and STRV, particularly because these viruses remained largely limited to Australia even after European colonization and consistent to the main distribution of macropods [151,152]. Horses could also be implicated in the transmission of KOKV as KOKV group-specific antibodies have been detected in Australian horses, and it is currently unknown whether viruses in the KOKV group is associated with known equine disease [64,65]. At this stage, all five members of the KOKV group will need further characterization and definition. Recently, the complete coding sequences of STRV, BAIV and Torres virus have become publicly available and is first step towards understanding the virus’ virulence [153].

## 11. Gan Gan and Trubanaman Viruses

Bunyaviruses are negative-stranded RNA viruses, consisting of three RNA segments which are named small, medium and large due to the length of nucleotides [154]. The Gan Gan virus (GGV) and Trubanaman virus (TRUV) were first isolated in 1966 (isolate MRM3630) [68] and 1970 (isolate NB6057) [67], respectively. Both viruses were only genetically characterized recently in 2016 [154]. Currently, GGV and TRUV have only been reported in Queensland, New South Wales and Western Australia [66].

GGV and TRUV cause polyarthritic illness, with symptoms similar to RRV. Because of its similarity to RRV, it is speculated that RRV-like infections that have negative serology for RRV may have been GGV or TRUV infections [154]. Generally, GGV neutralizing antibodies have higher titres and prevalences in humans compared to TRUV in most reported areas [26,70,155] except for Cape York Peninsula in Queensland [154]]. Humans are most likely a dead-end host as no horizontal or vertical transmissions have been reported [156]. There are currently no treatments for GGV or TRUV.

Bunyaviruses have multiple vectors that include arthropods, murids and bats; however, mosquitoes are the main vectors for GGV and TRUV [154]. Isolation of TRUV has been reported in *An. annulipes* [66] and *Cx. annulirostris* [62] mosquitoes, whereas GGV is most commonly isolated from *Ae. vigilax* [67], but has also been isolated in *Cx. annulirostris* [68] and *Anopheles meraukensis* [69].

In general, there is limited knowledge of the clinical signs and symptoms of GGV and TRUV and its effect on vertebrates, however serological studies have found antibodies in a broad range of mammals. A report in 1991 found GGV and TRUV antibodies in macropods, cattle, and horses in New South Wales [26]. Antibodies to GGV were also found in one out of 76 bush rats (*Rattus fuscipes)*, suggesting that the virus is able to infect murids [26]. Antibodies to TRUV have been found in western grey kangaroos, feral pigs, rabbits, European red foxes (*Vulpes vulpes*), quokkas (*Setonix brachyurus*), and horses [70]. Despite exposure across a broad range of vertebrate species, research has indicated that macropods are a key host for both viruses [26,70]. The wide range of species reported to be seropositive for these bunyaviruses may reflect the wide ranging host range of host mosquitoes in Australia [26,70].

## 12. A Changing Australia and Its Consequences

The infection, amplification and transmission of the pathogens mentioned are often affected by environmental and climatic change (see [157] for a review). A study collating 19 articles concerning the impact of climate change on RRV outbreaks demonstrated that the complex ecology, interactions between social and environmental factors, and climate change and socioeconomic development needs to be considered when trying to understand the ecology of RRV and prevent/reduce viral transmission [158]. Climate change can influence mosquito and wildlife distribution directly and/or indirectly by changing behaviors or movements. For example, while rainfall is predicted to decrease in certain areas of Australia, sea levels are predicted to increase which can potentially create another source of water for mosquito breeding [158]. This potential environmental change should be considered particularly for viruses that can be transmitted by various mosquito species. While the population of some mosquito species will decrease, others may consequentially thrive. Additionally, humans respond to climate change by altering their surroundings, which could influence the survival of wildlife and distribution of mosquito species depending on their ability to adapt. Human land-use change is one of the primary drivers of a range of infectious disease outbreaks and modifiers of the transmission of endemic infections [159]. Anthropophilic mosquito species such as *Aedes aegypti* often increase in response to urbanization, particularly taking advantage of man-made objects and preferentially feeding on human hosts [160].

Australia also has one of the highest extinction rates of mammalian fauna in the world [161,162]. The surviving Australian species are currently threatened by competition and predation from a range of introduced mammalian species, the low levels of conservation funding compared to other countries and the effects of climate change. The problem with losing biodiversity in Australia is that it can result in the loss of a “dilution effect”, which predicts that high host species richness can lower pathogen transmission [163]. This particularly applies to vectors that feed on multiple host species varying in their competence for a particular pathogen. For example, lower incidence of human WNV and Lyme disease has been observed in areas of the United States with greater host diversity [164,165]. Thus, continued population decline, and loss of species represents a significant public health threat in Australia.

There is also concern for the transmission of mosquito-borne diseases between countries. Other than migrating animal reservoirs such as birds and bats, increased human movements are now influencing mosquito and mosquito-borne disease distribution. Many mosquito species have been found to survive long-distance flights, including *Anopheles* mosquito species which transmits malaria [137,166]. Global travel and trade also enables the establishment of exotic zoonotic pathogens due to the availability of suitable vectors and hosts in many different countries [167]. It is therefore important to discuss the potential effects of such changes in Australia, particularly for future disease management purposes.

### 12.1. Climate and Its Significance to Australia’s Unique Vertebrate Communities

Climatically, higher temperatures have swept the whole of Australia and have created a dry landscape that is prone to bushfires. Bushfires have occurred in areas unaccustomed to fires, and are predicted to be more severe and frequent in the future [168]. Unexpected fires cause stress on wildlife, triggering immunosuppression, which increases the chances of infectious diseases [169]. Most recently in 2019, New South Wales, Queensland, South Australia and Victoria experienced intense bushfires [170], and undoubtedly have led to a decrease in wildlife populations [171]. Previous intense bushfires have caused devastating impacts on various marsupials including the koala [172], quokka [173], and possums [174].

Drought is a long term trend that is a natural part of the Australian hydroclimate; however, in addition to natural drought, the continuously changing agriculture and infrastructure landscape and societal context leads to a limited time to learn, adapt and prepare for droughts (see [175] for a review). While it is true that egg laying by mosquitoes decline during droughts, some mosquito species are able to retain their eggs for extended periods allowing them to search out remnant water sources during prolonged periods of drought [176]. Drought conditions may also increase the vector competence of *Cx. quinquefasciatus* for WNV by altering the immune response against the virus [177]. Increased temperatures associated with drought may also extend the length of disease transmission by increasing the normal seasonal activity of major vector species. For example in urban environments, *Culex* mosquitoes have been shown to breed earlier and extend their breeding season due to an increase in environmental temperatures [178]. Drought also leads to humans storing more water containers around houses, leading to increased mosquito breeding and disease outbreaks, particularly diseases associated with container-inhabiting species like dengue, chikungunya, and Zika [179,180].

Increased drought will also likely affect the abundance and distribution of competent vector species. In Australia, repeated drought events have decreased the survival and reproductive fitness of some smaller marsupials. After experiencing drought, the female agile antechinus (*Antechinus agilis*) survival and number of young per litter decreased and some females failed to give birth [181]. The brush-tailed phascogale (*Phascogale tapoatafa*) delayed births by increasing period of sperm storage beyond the drought and while beneficial, would decrease populations if the drought was long-term [182]. Bigger marsupials such as kangaroos change their distribution depending on drought and rainfall, with red kangaroo (*Macropus rufus*) populations moving long distances and aggregating at areas with a higher quality food supply and water [183]. Higher temperatures also lead to heat stress, which coincides with larger admissions of Australian birds and marsupials into veterinary clinics [184]. It is also important to mention that some Australian animals have a proven ability to cope with higher temperatures, however many also suffer from heat stress [185].

### 12.2. Climate and Its Significance to Mosquitoes

Climatic factors influence mosquito breeding and disease transmission [9]. The Australian continent has increased in temperature by 0.9 °C between 1910 to 2011, which is higher than the global temperature increase of 0.7 °C [186]. Predicted climate change in Australia will likely increase the distribution of Australian vector-borne diseases such as RRV [187]. Disease distribution also relies on the type of vector and its efficiency in spreading disease. For example, the *Ae. aegypti* population in Australia migrated from Western Australia, Northern Territory and New South Wales to Queensland, which led to repeated outbreaks of dengue [188]. Thus, the spread of disease will ultimately rely upon the distribution of suitable vector species. The distribution of the principal vectors of dengue, malaria, and other global vector-borne diseases are projected to increase considerably under current climate change scenarios as warming temperatures will allow them to spread to areas previously unsuitable for survival [189,190,191]. If correct, such increases will surely result in the spread of disease to previously uninfected areas. Although transmission of vector-borne diseases can be limited by seasonal temperature change in temperate environments [192], many mosquito species have proven highly adaptable to survive in areas of lower humidity [193] and even in areas that under winter [194].

Rising sea levels in response to climate change will dramatically change shoreline hydrology, causing marshes and seagrass beds to migrate landward, and will push salinity up the estuary [195]. The landward expansion of saline habitat may increase the risk of vector-borne disease outbreaks in many regions of the world by increasing the distribution of salt-associated mosquito species [196]. In Australia, the major salt marsh mosquitoes *Ae. vigilax* and *Ae. camptorhynchus* are important vectors of RRV and BFV [16] and any increase in their distributions represents a significant public health threat [197]. In addition to increased disease risk, the landward expansion of saline environments will increase the already extreme biting nuisance potential of these species [198,199] decreasing the quality of life in may coastal areas.

### 12.3. Urbanization and Habitat Fragmentation

Ecological change resulting from land-use modification often leads to the transmission of infectious diseases from wild animals to humans [200], and Australia is no exception. Vector-borne disease outbreaks from Australian wildlife almost always involve the installation of wetlands, encroachment of residential developments on reclaimed coastal wetlands or remote locations and deforestation (see [201] for a review). Although urbanization has led to the decline of certain marsupial populations due to decreased habitat [202,203], the impacts of such reductions on endemic arboviruses is not currently known.

Deforestation also leads to changes in wildlife movements, either away from the development, or adapting to human settlement. Whereas, urbanization decreases host species richness, as only some are capable of adaptation. Marsupials such as koalas have been historically and significantly affected by changing landscapes. As koalas are specialized feeders of predominantly *Eucalyptus* and *Corymbia* species, their diet also varies within regional areas due to different soil characteristics, tree structures, leaf water, and chemical content [204,205], which makes it difficult for koalas to adapt with the cumulative threats from environmental and landscape changes. They are also threatened by disease (i.e., chlamydial infections and koala retrovirus) and stress from habitat fragmentation or clearing, is expected to result in population decline (see [206] for a review). Some koala populations persist in urban landscapes where resources are available; however, patchy resources also increase their risk of death [207]. Reduced nutritional and population health likely compromise immunological fitness [208] and enhance the potential of some koala species to act as reservoirs or reduce the removal of certain pathogens from a host.

Even marsupials that are found in high densities in urban landscapes have been affected by urbanization and human population growth. The eastern grey kangaroo declined in overall population by 42% in south east Queensland, with a further decline anticipated with the increase of humans [202]. Additionally, land clearing and timber harvesting have also had an impact on the structure and distribution of various marsupial species because of the change in predation and food availability. For example, eastern grey kangaroos prefer the relatively open foraging sites for grazing and swamp wallabies prefer dense vegetation sites for feeding, suggesting that there will be changes of marsupial distribution depending on specific preferences of the species (see [209] for a review). Koalas from south east Queensland, Australia are more exposed to major RRV mosquito vector, *Cx. annulirostris*, because of their confinement to edges of permanent wetlands that are not suitable for urban development [89]. However, while some Australian wildlife species are struggling to adapt to rapid environmental and climatic change, some are proliferating. Possums, for example, have been seen thriving in urban environments as they are more tolerant of disturbances compared to other marsupials [203,210]. While this is good for the maintenance of the possum population, it is also suspected that they might cause disease outbreaks in urban areas due to the lack of biodiversity and the close proximity to humans or domestic animals [211].

## 13. Conclusions

This review summarises the current literature on mosquito-borne viruses in non-human species in Australia. Mosquito-borne viruses threaten both human and non-human vertebrate health; as such it is critical to periodically review the impacts of mosquito-borne pathogens in non-human species. Since Australian marsupials are considered key hosts for endemic mosquito-borne diseases such as RRV and BFV, these vertebrates could potentially be reservoirs for introduced mosquito-borne diseases. Alternatively, Australia’s diverse faunal species could prevent the spread of disease, reinforcing the importance of studying the role non-human vertebrates’ play in mosquito-borne disease transmission, although this diversity is currently under threat. We emphasise that it is important to consider the impact of non-human vertebrates on mosquito-borne diseases, particularly in mosquito control strategies and predicting future disease outbreaks. Environmental conditions in prediction studies are important, but there have been circumstances where they do not significantly affect the distribution of mosquitoes or contribute to disease outbreaks in Australia [212,213]. However, there is a possibility that the environment may influence animal behavior, further influencing the spread of disease. Understanding the complexity of factors that influence the transmission of mosquito-borne diseases will help us develop strategies to minimize the risk of outbreaks.

## Figures and Tables

**Table 1:** A summary of pathogen, pathogen distribution, mosquito vectors, and symptomatic ^a^ and asymptomatic ^b^ host distribution in Australia. Dengue and Rift Valley fever are not listed as there have been no known non-human vertebrate hosts in Australia.

Pathogen	Pathogen Distribution in Australia	Mosquito Vectors	Asymptomatic Hosts of Pathogen in Australia	Symptomatic Hosts of Pathogen in Australia
Ross River virus (RRV)	All of Australia [13]	*Aedes* and *Culex* mosquitoes, particularly *Aedes vigilax*, *Aedes camptohynchus*, and *Culex annulirostris* [14]	Marsupials: wallabies, wallaroos [15], common brushtail possums [16], eastern grey kangaroos [17], western grey kangaroos [18] Australian birds: little corella, magpie larks, Australian brown flycatcher, masked finch [19,20] Wild eutherian mammals: rodents, *Pteropus* spp. [20] Domestic mammals: cattle, dogs [20], cats [21]	Domestic mammals: horses [20,22]
Barmah Forest virus (BFV)	All of Australia [23]	*Culex annuliristris*, *Aedes normanensis*, *Aedes vigilax* and *Aedes procax* [24]	Marsupials: eastern grey kangaroo [25], koalas [25] and brushtail possums [21]. Wild eutherian mammals: Australian bush rats and swamp rats [26] Domestic mammals: cats, dogs, horses [27]	
Sindbis virus (SINV)	Most of Australia (excluding Tasmania) [28]	*Culex annulirostris*, *Aedes normanensis*, *Aedes camptorhynchus* [29], *Aedes pseudonormanensis* [30]	Marsupials: chudditch [31] Wild eutherian mammals: European rabbits [31] Domestic mammals: horses [31] Birds: Emus [31]	
Murray Valley encephalitis virus (MVEV)	Western Australia [32], Northern Territory [33], New South Wales, and Victoria [34]	*Culex annulirostris* [35], *Culex sitiens* and other *Culicine* mosquitoes [36]	Marsupials: eastern grey kangaroos [37], western grey kangaroos, agile wallabies [38] Australian birds: galahs, sulphur-crested cockatoos [38], chickens [39] Wild eutherian mammals: rabbits [37], wild mice [38] Domestic mammals: dogs, sheep, pigs, cattle [37] Water birds: rufous night herons [34], Pacific black ducks [38]	Domestic mammals: horses [22,40,41]
West Nile virus (WNV)	All of Australia [42,43]	Mainly isolated from *Culex annulirostris* [44]. Other *Culex* species, *Aedes* species and *Anopheles amictus* can also transmit the virus [45,46]	Marsupials: western grey kangaroos, agile wallabies [37] Australian bird: Australian white ibis [47] Ardeid birds: herons, egrets [48] Introduced bird: house sparrow [49].	Wild eutherian mammals: rabbits [50,51] Domestic mammals: horses [22,42], cats (mild) [52]
Japanese encephalitis virus (JEV)	Torres Strait (incursion) [53], North Peninsula Area and mainland [54]	*Culex annulirostris* [53].	Ardeid birds: herons, egrets [55,56,57] Domestic mammals: pigs [58], horses [59]	Wild eutherian mammals: Frugivorous bats i.e., black flying fox [60] Other birds: pigeons, sparrows^,^ ducks, chickens [60]
Kokobera (KOKV) and related viruses	Queensland, New South Wales, Northern Territory, Western Australia, and Papua New Guinea [61,62]	*Aedes* species including *Aedes aculeatus*, *Aedes alternas*, *Aedes notoscriptus*, *Aedes procax*, *Aedes vigilax* and *Anopheles annulipes* [63]	Marsupials: mainly kangaroos and wallabies [17,64]. Domestic mammals: cattle [26]	Domestic mammals: horses [64,65] It is unknown whether horses are affected by Kokobera and related viruses, as it could be associated with a known equine disease.
Gan Gan (GGV) and Trubanaman viruses (TRUV)	Queensland, New South Wales, and Western Australia [66]	*Aedes vigilax* (GGV) [67], *Culex annulirostris* (GGV and TRUV) [62,68], *Anopheles annulipes* (TRUV) [66] and *Anopheles meraukensis* [69]	Marsupials: eastern grey kangaroos (GGV, TRUV), red-necked wallaby (GGV, TRUV) [26], western grey kangaroos (TRUV) [70] Wild eutherian mammals: Australian bush rat (GGV) [26], feral pigs (TRUV), rabbits (TRUV), foxes (TRUV), quokkas (TRUV) [70] Domestic mammals: sheep (GGV), horses (GGV, TRUV), cattle (GGV) [26,70]	

^a^ A asymptomatic host would be a reservoir host species that does not experience the symptoms of disease but is able to produce sufficient pathogen levels to infect mosquitoes that blood-feed on that animal. ^b^ A symptomatic host would be a non-reservoir host species that show symptoms of the disease and also produces sufficient pathogen levels to infect mosquitoes that blood-feed on that animal.

## Data Availability

Data sharing not applicable.
